# Application of ^123^I-MIBG myocardial maximum standardized uptake value to characterize cardiac function in patients with pheochromocytoma: comparison with echocardiography

**DOI:** 10.1007/s11604-022-01365-z

**Published:** 2022-11-28

**Authors:** Masatoyo Nakajo, Yoshihisa Horizoe, Kodai Kawaji, Megumi Jinguji, Atsushi Tani, Yoshihiko Fukukura, Mitsuru Ohishi, Takashi Yoshiura

**Affiliations:** 1 Department of Radiology, Graduate School of Medical and Dental Sciences, Kagoshima University, 8-35-1 Sakuragaoka, Kagoshima, 890-8544 Japan; 2 Department of Cardiovascular Medicine and Hypertension, Graduate School of Medical and Dental Sciences, Kagoshima University, 8-35-1 Sakuragaoka, Kagoshima, 890-8544 Japan

**Keywords:** Pheochromocytoma, Cardiac function, [^123^I]metaiodobenzylguanidine, SPECT/CT, SUV

## Abstract

**Purpose:**

This study examined the usefulness of the maximum standardized uptake value (SUVmax) of myocardial [^123^I]-metaiodobenzylguanidine ([^123^I]-MIBG) to characterize myocardial function by comparing it with echocardiographic parameters in patients with pheochromocytoma.

**Materials and methods:**

This study included 18 patients with pheochromocytoma who underwent both planar and [^123^I]-MIBG single-photon emission computed tomography/computed tomography scans and echocardiography before surgery. Myocardial [^123^I]-MIBG visibility and SUVmax were compared with echocardiographic parameters related to systolic and diastolic functions. The Mann–Whitney U test, Fisher exact test, or Spearman rank correlation assessed differences or relationships between two quantitative variables.

**Results:**

On visual analysis, 6 patients showed normal myocardial [^123^I]-MIBG uptake, whereas 12 patients showed decreased myocardial [^123^I]-MIBG uptake. No patients showed systolic dysfunction. A significant difference was observed in the incidence of diastolic dysfunction between the groups with normal and decreased uptake (*p* = 0.009), and left ventricular (LV) diastolic dysfunction was observed in 9 (75%) of 12 patients with decreased myocardial uptake. The myocardial SUVmax was significantly lower in 9 patients with LV diastolic dysfunction than in 9 patients with normal cardiac function (1.67 ± 0.37 vs. 3.03 ± 1.38, *p* = 0.047). Myocardial SUVmax was positively correlated with septal e′ (early diastolic velocity of septal mitral annulus) (ρ = 0.51, *p* = 0.031) and negatively correlated with the septal E/e′ ratio (early mitral E-velocity to early diastolic velocity of septal mitral annulus; ρ =  − 0.64, *p* = 0.004), respectively.

**Conclusions:**

LV diastolic dysfunction was inversely related to myocardial [^123^I]-MIBG uptake. Myocardial [^123^I]-MIBG SUVmax may be useful for characterizing cardiac function in patients with pheochromocytoma.

Second abstract.

The semiquantitative analysis using the myocardial SUVmax in ^123^I-MIBG SPECT/CT was found to be potentially useful for characterizing cardiac function in patients with pheochromocytoma.

**Supplementary Information:**

The online version contains supplementary material available at 10.1007/s11604-022-01365-z.

## Introduction

In patients with pheochromocytoma (pheochromocytoma patients), cardiac complications due to an excess amount of catecholamines, including arrhythmias, Takotsubo-like cardiomyopathy, and dilated cardiomyopathy have been reported. These complications may be life-threatening. Hence, timely diagnosis and surgery can be life-saving [1].

Scintigraphy using iodine-123 metaiodobenzylguanidine ([^123^I]-MIBG), a radiolabeled noradrenaline analog, localizes pheochromocytomas and paragangliomas and estimates cardiac adrenergic activity, especially the function of presynaptic sympathetic nerve endings [2].

Several reports stated that semiquantitative analysis of [^123^I]-MIBG cardiac planar or single-photon emission computed tomography (SPECT) can predict the prognoses of patients with chronic heart failure [3–6]. However, estimating the uptake of local tracer using SPECT images can be technically complex, and inter- or intraobserver variability might not be negligible [6].

SPECT/ X-ray computed tomography (CT) (SPECT/CT) is a relatively new technology that enables the near-simultaneous coregistration of both functional and anatomical information that is acquired within the same imaging session with the patient in the same bed position [7]. The standardized uptake value (SUV), a semiquantitative parameter commonly used for positron emission tomography, has recently been applied to SPECT/CT [8, 9]. However, no studies have investigated the usefulness of SUV obtained from [^123^I]-MIBG cardiac SPECT/CT for characterizing cardiac function in pheochromocytoma patients.

This study aimed to examine the usefulness of [^123^I]-MIBG myocardial maximum SUV (SUVmax) on SPECT/CT by comparing it with echocardiographic function in pheochromocytoma patients.

## Materials and methods

### Patients

Our institutional review board approved this retrospective study. The need for informed consent was waived due to the retrospective nature of the analysis and the anonymity of the data. [^123^I]-MIBG planar and SPECT/CT scans were performed from April 2018 to August 2021 in 35 consecutive patients with suspected pheochromocytoma. We reviewed the clinical records to identify patients for analysis. The following inclusion criteria were applied: (1) pathological diagnosis of pheochromocytoma based on surgical specimens and (2) echocardiography performed before surgery. The exclusion criteria were (1) patients with other adrenal tumors; (2) patients with a history of neurological disorders such as Parkinson’s disease and Lewy body dementia, which could reduce myocardial [^123^I]-MIBG uptake; and (3) patients with a history of cardiac disorders such as myocardial infarction and dilated cardiomyopathy, except for catecholamine-induced heart disease, which the cardiovascular physicians determined.

We also reviewed the medication status because certain drugs, such as antihypertensive calcium-channel blockers, are known to reduce myocardial [^123^I]-MIBG uptake. Plasma catecholamine concentrations were measured before surgery and within two months of the [^123^I]-MIBG imaging (mean ± standard deviation [SD], 15 ± 14 days; range, − 36 to + 49 days).

### Imaging protocols for [^123^I]-MIBG SPECT/CT

Planar images of the anterior and posterior views and chest to abdominal SPECT/CT images were taken one day after intravenous injection of 111 MBq (3 mCi) of [^123^I]-MIBG (FUJIFILM Toyama Chemical Co., Ltd. Tokyo, Japan) using a dual-head gamma camera with medium-energy parallel-hole collimators and a multidetector (16-row) spiral CT (Siemens Intevo SPECT/CT system; Siemens Medical Solutions USA, Inc.). Planar images were acquired with a 256 × 1024 matrix and the photopeak energy window was 159 (± 15%) keV for [^123^I]. We acquired the SPECT/CT images with a matrix size of 128 × 128. A total image of 30 frames was acquired with an acquisition time of 30 s/frame and an angular step of 6°. After SPECT acquisition, we acquired CT images using a tube voltage of 130 kV and a dose-modulation algorithm with a quality reference mAs setting of 15 (CAREDose 4D; Siemens Medical Solutions USA, Inc.). The SPECT data were reconstructed with attenuation and scatter correction using a three-dimensional iterative algorithm (Ordered Subset Conjugate-Gradient Minimizer; Siemens Medical Solutions USA, Inc.).

### Phantom study for SUV

We performed a phantom experiment to calculate the cross-calibration factor (CCF) for converting SPECT count images to SPECT SUV images. The radioactivity of the [^123^I]-MIBG syringe was measured before injection of [^123^I]-MIBG into the cylindrical phantom (20 cm in diameter and 20 cm in height; Anzai Medical, Tokyo, Japan) with the measured time recorded. The [^123^I]-MIBG was injected into the cylindrical phantom, and the time of the [^123^I]-MIBG injection was recorded. The radioactivity of the [^123^I]-MIBG syringe was also measured after [^123^I]-MIBG injection while recording the measured time. After that, we scanned the cylindrical phantom in which 109.5 MBq of [^123^I]-MIBG was diluted uniformly by adding water, recorded the scan starting time, and reconstructed the images according to the above clinical protocol. We calculated the CCF using a workstation (*syngo* MI Workplace; Siemens Medical Solutions USA, Inc.).

### Analysis of [^123^I]-MIBG images

The [^123^I]-MIBG planar, SPECT, and SPECT/CT images were displayed on a workstation (Syngo.via; Siemens Healthcare GmbH, Erlangen, Germany) and reviewed in consensus by two experienced radiologists who knew the study purpose but were blinded to the patients’ clinical information. The observers assessed the myocardial and adrenal pheochromocytoma [^123^I]-MIBG uptake on the planar images using a score scale as follows: 0, no uptake; 1, faint uptake less than that of the liver; 2, uptake equal to that of the liver; and 3, uptake greater than that of the liver [10]. Scores of 2 to 3 were considered as normal myocardial uptake, and scores of 0 to 1 were considered to indicate decreased myocardial uptake, respectively. To determine the presence or absence of [^123^I]-MIBG uptake in the pheochromocytomas, scores of 0 to 1 and 2 to 3 were nonvisible and visible, respectively.

The following semiquantitative analyses according to the interpreted results of the visual assessment were performed using the dedicated software (Syngo.via) by one of the above observers and another experienced radiologist, independently. The heart-to-mediastinum (H/M) ratio was calculated from the anterior planar [^123^I]-MIBG image using the regions of interest (ROIs) over the heart and the upper part of the mediastinum. The cardiac ROI was drawn manually over the heart, including the left ventricular (LV) cavity. The mediastinal ROI was also placed manually over the upper part of the mediastinum. The H/M ratio was calculated by dividing the mean counts per pixel in the cardiac ROI by the mean counts per pixel in the mediastinal ROI [11]. On the quantitative analysis for H/M ratio, the above ROI setting was performed twice with an interval of one week between measurements for each observer, and the averaged cardiac or mediastinal ROI size for each time for each observer is shown in the supplemental table 1.

The SUVmax values of the myocardium, liver and adrenal pheochromocytoma were recorded as follows. First, the volume of interest (VOI) was drawn manually around the heart, liver and the pheochromocytoma in a suitable reference transaxial plane, excluding the adjacent avid noncardiac, nonhepatic, or nonpheochromocytoma structures using the CT images as the reference, respectively. We defined the SUVmax as the maximum tissue concentration in the structure delineated by the VOI divided by the activity injected per gram of body weight. On the quantitative analysis for SUV-related parameter, the above VOI setting was performed twice with an interval of one week between measurements for each observer.

The measured values for each H/M ratio or SUV-related parameters (myocardial SUVmax, liver SUVmax, pheochromocytoma SUVmax) were averaged to represent the results of each parameter.

### Echocardiographic study

Conventional routine transthoracic echocardiography (TTE) was performed before surgery within three months of [^123^I]-MIBG imaging (mean ± SD, 25 days ± 24; range, − 8 to + 83 days). TTE was carried out using an IE33 system (Phillips Medical Systems, Andover, MA, USA) by a highly experienced cardiologist who was unaware of the purpose of the study. The ejection fraction (EF) was assessed using the Simpson’s modified method.

To obtain the mitral inflow pattern, we obtained pulsed-wave Doppler echocardiography recordings from a sample volume positioned at the tips of the mitral valves parallel to inflow during diastole at end-expiration. The following parameters were measured to evaluate the diastolic function of the left ventricular (LV): peak early filling (E) and its deceleration time, atrial filling peak (A), and the E/A ratio. The moving velocity of the septal LV wall was also assessed by tissue Doppler imaging (TDI) through the apical window at apical four-chamber views. The sample volume was placed at the basal portion of the septal LV walls to determine the longitudinal expansion velocities, which were obtained by measuring the initial (e′) and final (a′) diastolic velocities. The septal E/e′ ratio was calculated by dividing the peak early wave of the transmitral flow (septal E) to the initial diastolic wave (septal e′) by TDI. We evaluated LV function based on echocardiographic findings, and normal ventricular function was defined as an EF > 50% with normal LV diastolic function (left atrial volume index [LAVI] < 28 mL/mm^2^, e′ normal for age [age < 55 years: e′ ≥ 10 cm/s, age 55–65 years: e′ ≥ 9 cm/s: age > 65 years: e′ ≥ 8 cm/s], E/A ratio > 0.8, and septal E/e′ ratio < 8) [12].

### Statistical analysis

The agreement between the results (intra- and inter-observer variability) was measured using intra- or inter-class correlation coefficient (ICC) and Bland and Altman analysis. The ICC was reported with a 95% confidence interval where ICC < 0.4 was considered poor agreement, ICC 0.40–0.59 was considered fair agreement, ICC 0.60–0.74 was considered good agreement, and ICC > 0.74 was considered excellent agreement [13]. Bland–Altman analyses were expressed as the mean difference and 95% limits of agreement [14]. Lower and upper reproducibility limits, defining the reference range of spontaneous changes, were calculated as ± 1.96 × SD, which provided that the distribution was not statistically different from a normal one.

We used the Mann–Whitney *U* test or Fisher exact test to assess the difference between the two quantitative variables or compare categorical data. Spearman rank correlation was used to assess the relationship between two quantitative variables.

Data were expressed as the mean value ± SD and/or range. A *p* < 0.05 value was considered statistically significant, and all *p*-values presented were two-sided. We performed statistical analyses using MedCalc Statistical Software (MedCalc Software, Mariakerke, Belgium).

## Results

Of the 35 patients, there were 17 patients (8 men, 9 women; mean [± SD] age, 66 ± 12 years; range, 40–83 years) without pheochromocytoma (nonpheochromocytoma patients); 4 patients did not have adrenal tumors, and 13 patients had the tumors other than pheochromocytoma (n = 8 cortical adenoma, n = 2 metastatic adrenal tumors [one melanoma and one renal cell carcinoma], one myelolipoma, one hemangioma and one ganglioneuroma). No significant difference was observed in age between the pheochromocytoma and nonpheochromocytoma patients (*p* = 0.15; supplemental Table 2). Each plasma catecholamine level was significantly higher in the pheochromocytoma patients than in the nonpheochromocytoma patients (each, *p* < 0.05; supplemental Table 2). The comparisons of the myocardial visual score and quantitative parameters including myocardial SUVmax, H/M raio and liver SUVmax were performed between the pheochromocytoma and nonpheochromocytoma patients as the supplemental analysis. Upon visual analysis of myocardial [^123^I]-MIBG uptake, all nonpheochromocytoma patients showed normal myocardial [^123^I]-MIBG uptake (all visual scores 2), and the myocardial visual score was significantly lower in the pheochromocytoma patients than in the nonpheochromocytoma patients (*p* < 0.001) (supplemental Table 2). Both the myocardial SUVmax and H/M ratio were significantly lower in the pheochromocytoma patients than in the nonpheochromocytoma patients (each, *p* < 0.05), while no significant difference in the liver SUVmax was observed between them (*p* = 0.08) (supplemental Table 2). None of the 35 patients had a history of neurologic or cardiac disorders that could reduce myocardial [^123^I]-MIBG uptake, and was being treated with labetalol (alpha–beta-blocker) known to reduce [^131^I]-MIBG uptake of the liver [15]. These 17 nonpheochromocytoma patients were excluded from the following analyses of pheochromocytoma patients.

Finally, 18 patients (11 men, 7 women; mean [± SD] age, 58 ± 16 years; range, 28–76 years) were eligible for the analyses. None of the patients was being treated with drugs known to reduce myocardial [^123^I]-MIBG uptake, except for antihypertensive calcium-channel blockers, which could reduce [^123^I]-MIBG release from and increase its retention in nerve terminals [16]. All patients were treated for hypertension with antihypertensive drugs: 11 patients took alpha-blockers, three took calcium-channel blockers, two took alpha-blockers and calcium-channel blockers, one took angiotensin II receptor blockers, and one took diuretics.

Based on the echocardiographic findings, 9 patients showed normal LV function and 9 patients showed LV diastolic dysfunction. The mean age was significantly higher in the LV diastolic dysfunction patients than in the LV normal function patients (68 years ± 7, range, 54–76 years, vs. 48 years ± 17, range, 28–75 years, *p* = 0.025). There were no significant differences in heart rate (HR) and systolic and diastolic blood pressure (BP) between the normal cardiac function and LV diastolic dysfunction patients (normal cardiac function vs. LV diastolic dysfunction: HR, 70 ± 10 beats/min vs. 64 ± 13 beats/min, *p* = 0.18; systolic BP, 115 ± 13 mmHg vs. 107 ± 7 mmHg, *p* = 0.14; diastolic BP, 72 ± 13 mmHg vs. 65 ± 5 mmHg, *p* = 0.23).

### Relationship between myocardial [^123^I]-MIBG uptake and cardiac function

Upon visual analysis of myocardial [^123^I]-MIBG uptake, 6 patients showed normal myocardial [^123^I]-MIBG uptake (all visual scores 2), whereas 12 patients showed decreased myocardial [^123^I]-MIBG uptake (all visual scores 1). No significant differences were observed in liver SUVmax (*p* = 0.16) and age (*p* = 0.22) between the normal and decreased [^123^I]-MIBG myocardial uptake groups (Table 1). We found no significant differences in medication with calcium-channel blockers between the group with decreased myocardial uptake (2/12, 16.7%) and those with normal myocardial uptake (3/6, 50%, *p* = 0.27). None of the 18 pheochromocytoma patients showed LV systolic dysfunction. We observed a significant difference in the presence of LV diastolic dysfunction between the groups with normal (0/6, 0%) and decreased myocardial (9/12, 75%) uptake (*p* = 0.009; Table 1).

**Table 1:** Comparison of age, plasma catecholamines, echocardiographic parameters, and ^123^I-MIBG scintigraphic parameters between decreased and normal myocardial uptake pheochromocytoma patients

Parameter	Decreased myocardial uptake (n = 12)	Normal myocardial uptake (n = 6)	
	Mean value ± SD (range)	Mean value ± SD (range)	*p* value
Age	62 ± 12 (41–76)	49 ± 21 (28–75)	0.22
Cardiac function	n	n	*p* value
LV diastolic dysfunction	9	0	0.009
Normal cardiac function	3	6	
Catecholamine*	Mean value ± SD (range)	Mean value ± SD (range)	*p* value
Adrenaline (pg/mL)	83.4 ± 67.1 (15.0–216.0)	140.2 ± 124.0 (19.0–345.0)	0.39
Noradrenaline (pg/mL)	1780.0 ± 1665.7 (233.2–5639.0)	931.0 ± 650.0 (441.0–2185.0)	0.51
Dopamine (pg/mL)	130.9 ± 228.5 (7.0–701.3)	13.3 ± 5.4 (6.0–22.0)	0.028
Echocardiography	Mean value ± SD (range)	Mean value ± SD (range)	*p* value
LVESD (mm)	27.8 ± 3.8 (22.7–36.6)	27.2 ± 3.9 (23.0–33.7)	0.78
LVEDD (mm)	47.2 ± 4.4 (40.0–52.6)	47.0 ± 4.1 (40.5–51.8)	0.93
IVST (mm)	10.8 ± 0.8 (9.4–11.4)	10.4 ± 2.1 (7.0–12.7)	0.74
PWT (mm)	10.7 ± 1.2 (8.3–12.0)	10.2 ± 1.8 (8.1–12.4)	0.78
EF (%)	68.0 ± 7.8 (58.8–80.7)	70.6 ± 8.2 (60.0–80.4)	0.51
LAD (mm)	40.4 ± 5.6 (31–47)	33.4 ± 5.6 (26–37)	0.028
LAVI (mL/mm^2^)	32.0 ± 10.8 (18.2–52.4)	25.0 ± 7.5 (21.1–27.6)	0.22
E (cm/s)	73.1 ± 18.9 (45.0–108.0)	67.7 ± 11.3 (46.7–80.0)	0.51
A (cm/s)	72.0 ± 23.4 (34.0–115.7)	67.8 ± 18.4 (37.8–85.8)	0.48
E/A	1.12 ± 0.49 (0.72–2.45)	1.15 ± 0.36 (0.86–1.81)	0.26
DT (ms)	208.8 ± 47.0 (130.0–320.0)	229.7 ± 72.7 (180.0–373.0)	0.89
Septal e′ (cm/sec)	6.8 ± 2.4 (4.0–10.0)	9.7 ± 1.4 (8.0–11.2)	0.013
Septal E/e′	11.0 ± 3.2 (7.7–17.1)	6.5 ± 0.9 (5.4–7.8)	0.001
^123^I-MIBG scintigraphy	Mean value ± SD (range)	Mean value ± SD (range)	*p* value
Liver SUVmax	3.95 ± 1.0 (2.37–5.97)	5.71 ± 2.4 (3.10–8.50)	0.16
Myocardial SUVmax	1.62 ± 0.38 (1.16–2.49)	3.80 ± 0.89 (2.62–4.75)	0.001
H/M ratio	1.64 ± 0.33 (1.15–2.14)	2.31 ± 0.28 (1.90–2.66)	0.003
Pheochromocytoma SUVmax	24.26 ± 25.64 (2.37–86.23)	6.32 ± 2.70 (2.79–9.01)	0.11

*A* velocity of late mitral filling, *DT* deceleration time of early mitral filling, *E*-velocity of early mitral filling, *E/A* ratio of early to late mitral filling velocities, *EF* ejection fraction, *H/M ratio* heart-to-mediastinum ratio, *IVST* interventricular septum thickness (diastole), *LAD* left atrium diameter, *LAVI* left atrial volume indexed to BSA, *LVEDD* left ventricular end-diastolic diameter, *LVESD* left ventricular end-systolic diameter, *PWT* posterior wall thickness (diastole), *SD* standard deviation, *septal e′* early diastolic velocity of septal mitral annulus, *septal E/e′* ratio of early mitral E-velocity to early diastolic velocity of septal mitral annulus, *SUVmax* maximum standardized uptake value

*Reference ranges for peripheral vein supine samples were adrenaline, < 100 pg/mL; noradrenaline, 100–450 pg/mL; and dopamine, ≤ 20 pg/mL

There was no significant difference in LVEF between the groups with normal and decreased myocardial uptake (*p* = 0.51; Table 1). With regard to the echocardiographic parameters related to LV diastolic function, the decreased myocardial uptake group showed significantly higher left atrial diameter (LAD), lower septal e′, and higher septal E/e′ ratio compared to the normal myocardial uptake group (each *p* < 0.05; Table 1). There were no significant differences in other echocardiographic parameters between the groups with normal and decreased myocardial uptake (each *p* > 0.05; Table 1).

The results of the semiquantitative analyses of myocardial [^123^I]-MIBG uptake showed significant correlations between myocardial SUVmax and visual score (ρ = 0.82, *p* < 0.001), between myocardial SUVmax and H/M ratio (ρ = 0.82, *p* < 0.001), and between H/M ratio and visual score (ρ = 0.73, *p* < 0.001). Neither myocardial SUVmax nor H/M ratio was significantly correlated with age (myocardial SUVmax, ρ =  − 0.10, *p* = 0.69; H/M ratio, ρ =  − 0.04, *p* = 0.88).

Both myocardial SUVmax and H/M ratio were significantly lower in the group with decreased myocardial uptake than in those with normal myocardial uptake (each *p* < 0.05; Table 1). The myocardial SUVmax was significantly lower in the group with LV diastolic dysfunction than in the group with normal cardiac function (*p* = 0.047), although no significant difference was observed in the H/M ratio between the groups with normal cardiac function and LV diastolic dysfunction (*p* = 0.33; Table 2).

**Table 2:** Comparison of ^123^I-MIBG scintigraphic parameters between LV diastolic dysfunction and normal cardiac function pheochromocytoma patients

Parameter	LV diastolic dysfunction (n = 9)	Normal cardiac function (n = 9)	*p* value
	Mean ± SD (range)	Mean ± SD (range)	
Myocardial SUVmax	1.67 ± 0.37 (1.27–2.49)	3.03 ± 1.38 (1.16–4.75)	0.047
H/M ratio	1.76 ± 0.29 (1.29–2.14)	1.96 ± 0.56 (1.15–2.66)	0.33
Pheochromocytoma SUVmax	23.80 ± 27.33 (4.34–86.23)	12.75 ± 15.93 (2.37–52.03)	0.23

*H/M ratio* heart-to-mediastinum ratio, *LV* left ventricular, *SD* standard deviation, *SUVmax* maximum standardized uptake value

Regarding the correlations between myocardial SUVmax and echocardiographic parameters, we observed a significant positive correlation between myocardial SUVmax and septal e′ (ρ = 0.51, *p* = 0.031), whereas we found significant negative correlations between myocardial SUVmax and LAD (ρ =  − 0.49, *p* = 0.041) and between myocardial SUVmax and the septal E/e′ ratio (ρ =  − 0.64, *p* = 0.004). No significant correlations were observed between myocardial SUVmax and the other echocardiographic parameters (each *p* > 0.05). We also found no significant correlation between the H/M ratio and any echocardiographic parameter (each *p* > 0.05; Table 3).

**Table 3:** Correlations of age, plasma catecholamines and echocardiographic parameters with ^123^I-MIBG scintigraphic parameters in 18 pheochromocytoma patients

Parameter	Myocardial SUVmax		H/M ratio		Pheochromocytoma SUVmax	
	Correlation		Correlation		Correlation	
	ρ	*p* value	ρ	*p* value	ρ	*p* value
Age	− 0.10	0.69	− 0.04	0.88	0.15	0.56
Catecholamine						
Adrenaline	0.42	0.086	0.41	0.089	− 0.21	0.39
Noradrenaline	− 0.24	0.33	− 0.37	0.13	0.42	0.083
Dopamine	− 0.44	0.071	− 0.48	0.044	0.02	0.95
Echocardiography						
LVESD	− 0.16	0.54	− 0.17	0.50	0.09	0.73
LVEDD	0.06	0.80	0.15	0.56	− 0.001	0.99
IVST	− 0.08	0.75	0.04	0.89	− 0.15	0.54
PWT	− 0.05	0.82	0.14	0.57	− 0.21	0.41
EF	0.32	0.20	0.42	0.086	0.09	0.72
LAD	− 0.49	0.041	− 0.39	0.11	0.18	0.47
LAVI	− 0.18	0.48	− 0.13	0.60	0.25	0.32
E	− 0.007	0.98	− 0.09	0.73	0.29	0.25
A	− 0.006	0.98	0.02	0.94	0.29	0.24
E/A	0.15	0.56	0.07	0.78	− 0.13	0.62
DT	− 0.01	0.96	0.02	0.94	− 0.34	0.17
Septal e′	0.51	0.031	0.44	0.07	− 0.32	0.19
Septal E/e′	− 0.64	0.004	− 0.38	0.12	0.44	0.066

*A* velocity of late mitral filling, *DT* deceleration time of early mitral filling, *E* velocity of early mitral filling, *E/A* ratio of early to late mitral filling velocities, *EF* ejection fraction, *H/M ratio*, heart-to-mediastinum ratio; *IVST* interventricular septum thickness (diastole), *LAD* left atrium diameter, *LAVI* left atrial volume indexed to BSA, *LVEDD* left ventricular end-diastolic diameter, *LVESD* left ventricular end-systolic diameter, *PWT* posterior wall thickness (diastole), *septal E/e′* ratio of early mitral E-velocity to early diastolic velocity of septal mitral annulus e′, *septal e′* early diastolic velocity of septal mitral annulus, *SUVmax* maximum standardized uptake value

**Fig.1:**
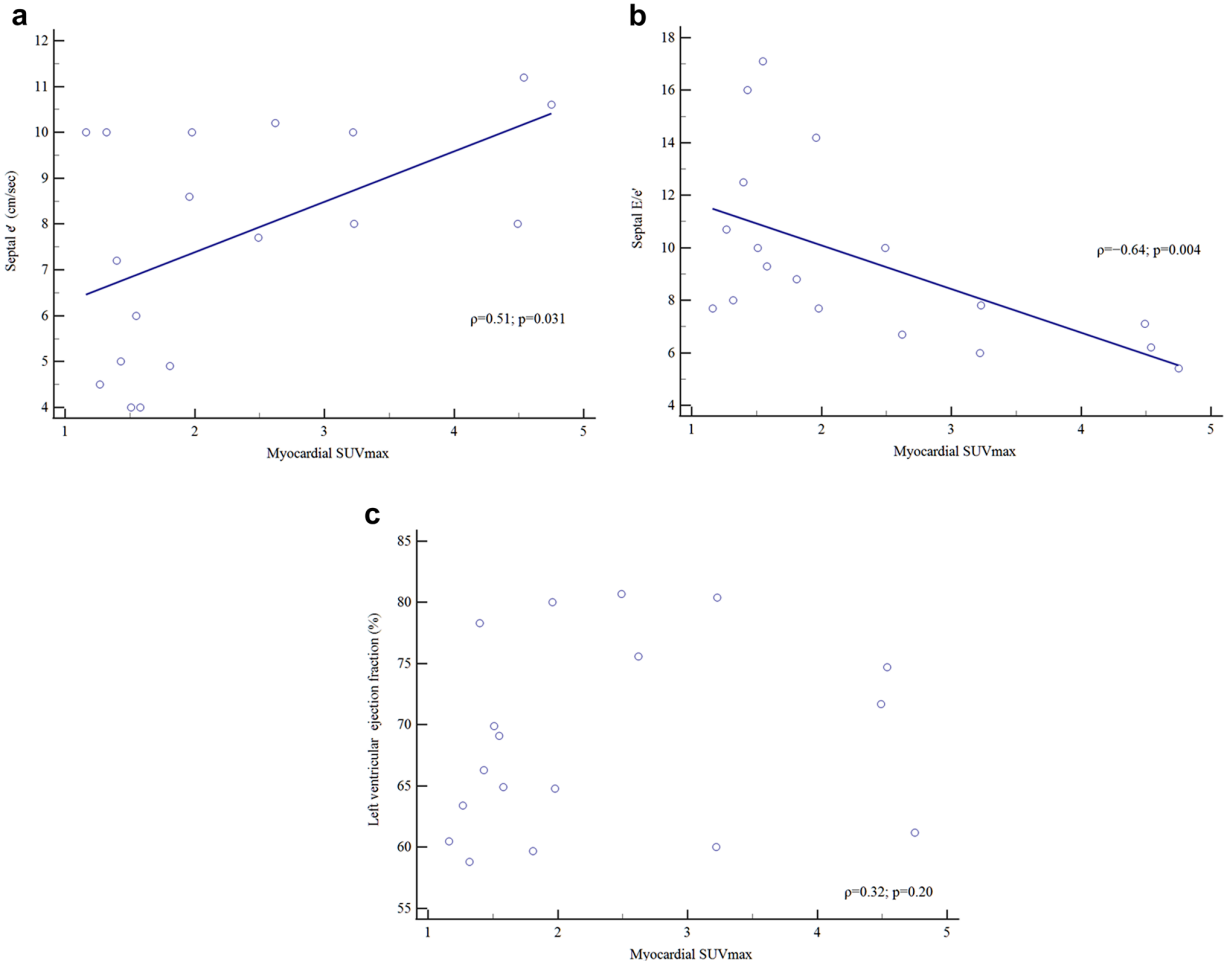
Scatter plots between myocardial SUVmax and septal e′ (**a**), myocardial SUVmax and septal E/e′ ratio (**b**), and myocardial SUVmax and LVEF (**c**). There are a significant positive correlation between myocardial SUVmax and septal e′ (ρ = 0.51, *p* = 0.031) (**a**), and a negative correlation between myocardial SUVmax and the septal E/e′ ratio (ρ =  − 0.64, *p* = 0.004) (**b**). No significant correlation is observed between myocardial SUVmax and the LVEF(ρ = 0.32, *p* = 0.20) (**c**).

Scatter plots between myocardial SUVmax and septal e′, septal E/e′ ratio, or LVEF are presented in Figs. 1a, 1b and 1c, resepectively.

**Fig. 2:**
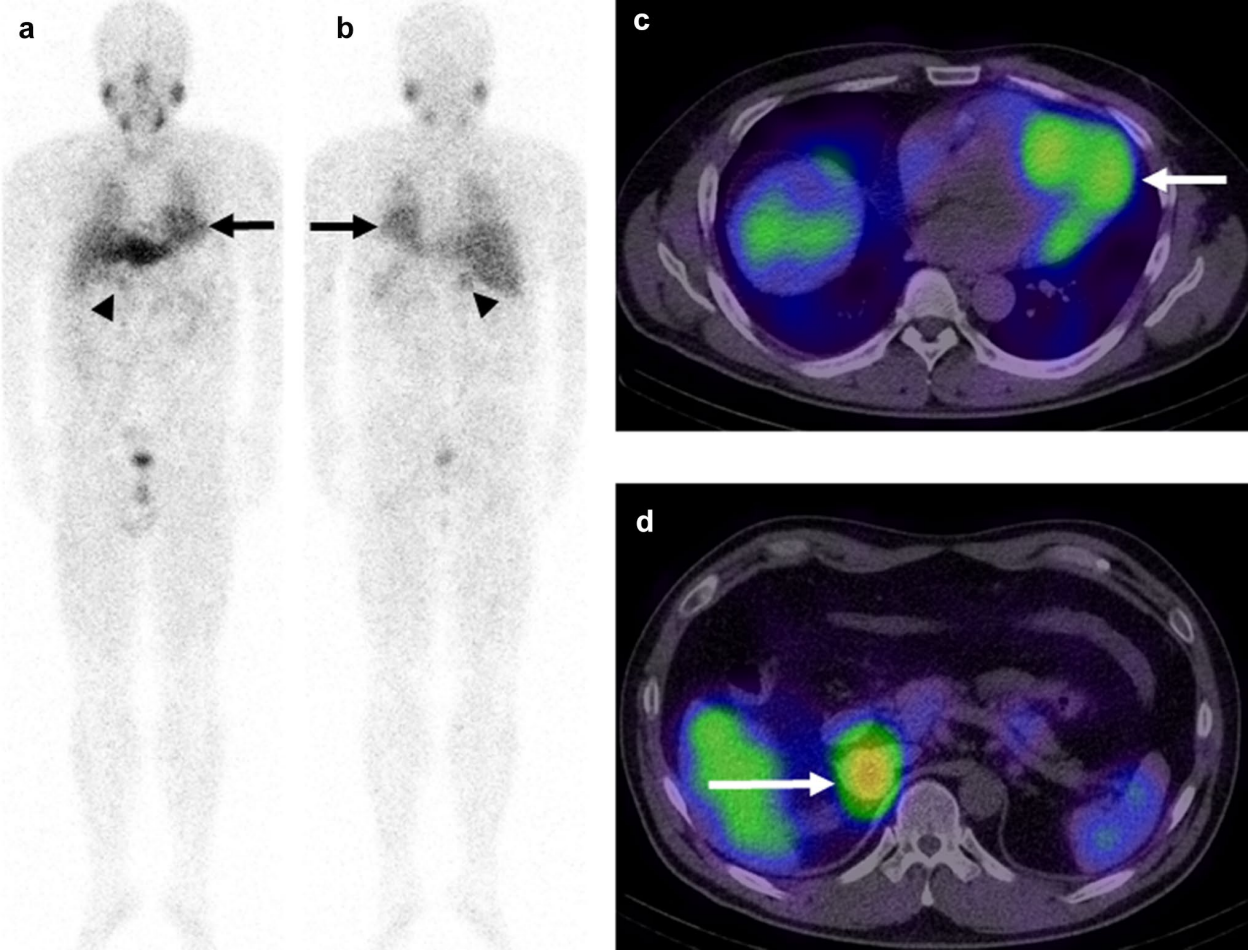
A 50-year-old male patient with right adrenal pheochromocytoma with normal cardiac function. The [^123^I]-MIBG anterior (**a**) and posterior (**b**) planar images show the normal cardiac uptake (arrow, score 2) and the pheochromocytoma uptake (arrowhead, score 2). The [^123^I]-MIBG SPECT/CT images show the [^123^I]-MIBG uptake in both the myocardium (**c**, arrow) and pheochromocytoma (**d**, arrow). The myocardium SUVmax, H/M ratio, and pheochromocytoma SUVmax were 4.54, 2.66, and 6.19, respectively. The LVEF, LAVI, E/A, septal e′, and septal E/e′ ratio were 74.7%, 23.2 mL/mm^2^, 0.97, 11.2 cm/s, and 6.2, respectively. These echocardiographic findings indicate normal cardiac function

**Fig. 3:**
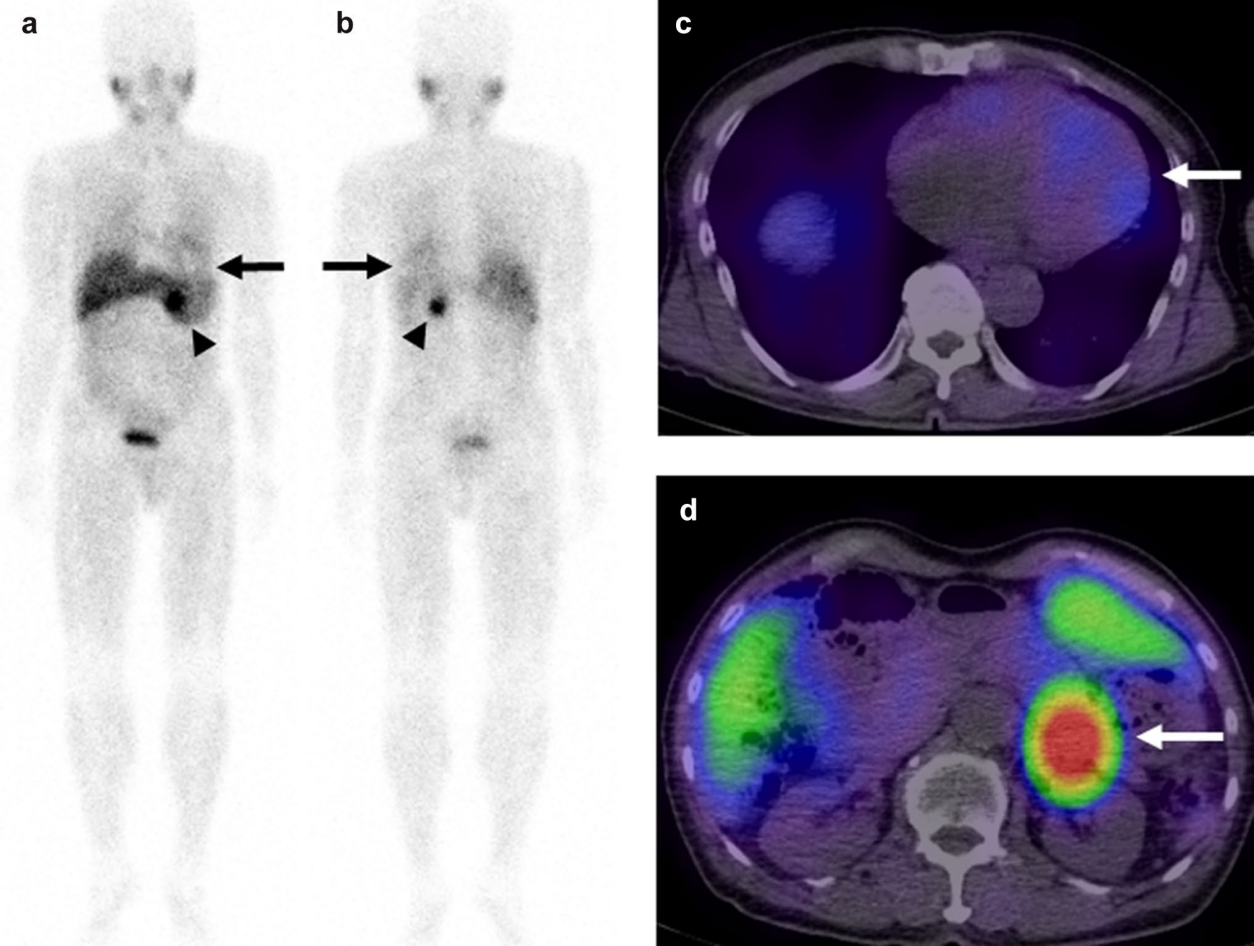
A 65-year-old male patient with left adrenal pheochromocytoma with LV diastolic dysfunction. The [^123^I]-MIBG anterior (**a**) and posterior (**b**) planar images reveal decreased cardiac uptake (arrow, score 1) and intense pheochromocytoma uptake (arrowhead, score 3). The [^123^I]-MIBG SPECT/CT images show slight [^123^I]-MIBG uptake in the myocardium (**c**, arrow), and intense [^123^I]-MIBG uptake in the pheochromocytoma (**d**, arrow). The myocardium SUVmax, H/M ratio, and pheochromocytoma SUVmax were 1.96, 1.98, and 15.47, respectively. The LVEF, LAVI, E/A, septal e′, and septal E/e′ ratio were 80.0%, 40.6 mL/mm^2^, 0.72, 4.0 cm/s, and 14.2, respectively. These echocardiographic findings indicate the presence of LV diastolic dysfunction

Representative [^123^I]-MIBG images of normal and decreased myocardial uptake pheochromocytoma patients are presented in Figures. 2 and 3, respectively.

### Relationships between myocardial [^123^I]-MIBG uptake and plasma catecholamine levels

The plasma dopamine level was significantly higher in the group with decreased myocardial uptake than in the group with normal myocardial uptake (*p* = 0.028; Table 1). There were no significant differences in plasma adrenaline or noradrenaline levels between the groups (each *p* > 0.05; Table 1).

We observed no significant correlations between plasma catecholamine levels and myocardial SUVmax (each *p* > 0.05; Table 3). However, we found a significant negative correlation between plasma dopamine levels and the H/M ratio (ρ =  − 0.48, *p* = 0.044), whereas other plasma catecholamine levels were not significantly correlated with the H/M ratio (each *p* > 0.05; Table 3).

The relationships between myocardial [^123^I]-MIBG uptake and plasma catecholamine levels among the 35 patients including the pheochromocytoma and nonpheochromocytoma patients were also performed as the supplemental analysis. In the 17 nonpheochromocytoma patients, plasma catecholamine concentrations were also measured within two months of the [^123^I]-MIBG imaging (mean ± SD, 25 ± 15 days; range, 4 to 49 days). Each myocardial [^123^I]-MIBG uptake parameter including myocardial visual score, myocardial SUVmax or H/M ratio was significantly and negatively correlated with plasma noradrenaline or dopamine levels (each *p* < 0.05; supplemental table 3), whereas plasma adrenaline levels were not significantly correlated with the above myocardial [^123^I]-MIBG uptake parameter (each *p* > 0.05; supplemental table 3).

### Relationships between pheochromocytoma [^123^I]-MIBG uptake and cardiac function and between pheochromocytoma and myocardial SUVmax

All pheochromocytomas were visualized by [^123^I]-MIBG scintigraphy. No significant difference was observed in the pheochromocytoma visual score between the group with normal cardiac function (2.6 ± 0.5, range:2–3) and the group with LV diastolic dysfunction (2.9 ± 0.3, range:2–3; *p* = 0.13) or between the normal myocardial (2.5 ± 0.5, range:2–3) and decreased myocardial (2.8 ± 0.4, range:2–3) uptake groups (*p* = 0.15). We observed no significant difference in the pheochromocytoma SUVmax between the groups with normal myocardial and decreased myocardial uptake (*p* = 0.11; Table 1) or between the normal cardiac function and LV diastolic dysfunction groups (*p* = 0.23; Table 2).

Neither catecholamine levels nor echocardiographic parameters significantly correlated with the pheochromocytoma SUVmax (*p* > 0.05; Table 3). There was no significant correlation between pheochromocytoma SUVmax and myocardial SUVmax (ρ =  − 0.28, *p* = 0.27).

### Intra- and inter-observer variability

The intraclass correlation coefficient (ICC)s and Bland–Altman analysis for intra-observer variability for cardiac ROI size, mediastinal ROI size, H/M ratio and all SUV-related parameters (myocardial SUVmax, liver SUVmax, pheochromocytoma SUVmax) in 35 patients are summarized in the supplemental table 4. Intra-observer agreement of each observer 1 or 2 was perfect for pheochromocytoma SUVmax and excellent or perfect for cardiac ROI size, mediastinal ROI size, H/M ratio, myocardial SUVmax and liver SUVmax. Bland–Altman plots of the cardiac ROI size, mediastinal ROI size, H/M ratio, myocardial SUVmax and liver SUVmax of each observer are presented in supplemental Figs. 1 and 2, and the Bland–Altman analysis showed no systmematic differences between assessments.

Inter-observer agreements were perfect for pheochromocytoma SUVmax, excellent for H/M ratio, myocardial SUVmax, and liver SUVmax and good for cardiac ROI size and mediastinal ROI size between observers 1 and 2 (supplemental table 5).

## Discussion

The current study evaluated the usefulness of myocardial [^123^I]-MIBG SUVmax for characterizing cardiac function by comparing it with echocardiographic parameters in pheochromocytoma patients. We found that LV diastolic dysfunction was inversely related to myocardial [^123^I]-MIBG uptake, as assessed by myocardial SUVmax. Therefore, the myocardial [^123^I]-MIBG SUVmax might have the potential to be an imaging biomarker for predicting diastolic dysfunction in patients with pheochromocytoma.

[^123^I]-MIBG cardiac scintigraphy assesses the integrity and activity of cardiac sympathetic neuronal innervation [2]. In addition, cardiac sympathetic nerve activity, as measured by cardiac MIBG uptake, has been an excellent discriminator of the prognosis of patients with heart failure [3]. The H/M ratio on the anterior planar image has been widely used as the semiquantitative myocardial [^123^I]-MIBG uptake parameter [17, 18]. A prior study reported that the semiquantitative analysis of [^123^I]-MIBG cardiac SPECT is useful for evaluating regional sympathetic activity [6, 19]. However, the disadvantage of semiquantitative analysis of [^123^I]-MIBG cardiac SPECT is its poor reproducibility [6, 19]. Recently, a semiquantitative parameter has introduced SUV derived from SPECT/CT imaging. SPECT/CT can generate imaging voxels denoted in units of radioactivity per volume (i.e., kBq/mL) using CT-based attenuation correction and scatter correction [20]. Therefore, by normalizing the lesion radioactivity for the injected radioactivity, SUV can be obtained [20, 21]. This technique differs from conventional nuclear imaging techniques, such as planar scintigraphy and SPECT, which use the counts per pixel for their imaging units.

Suga et al. [11] evaluated myocardial [^123^I]-MIBG (or [^131^I]-MIBG) uptake before and after treatment in patients with neuroadrenergic tumors (pheochromocytomas and neuroblastomas). The authors found no significant correlation between the 24-h H/M ratio and LVEF before or after treatment. Elenkova et al. [22] examined the echocardiographic findings of pheochromocytoma patients. They reported that LVEF was normal in all participants, and the pheochromocytoma patients showed LV diastolic dysfunction with a significantly lower septal e′ wave and a higher septal E/e′ ratio than patients with essential hypertension.

Our study found no significant difference in LVEF between the groups with normal and decreased myocardial uptake and no significant correlation between LVEF and myocardial SUVmax nor between the LVEF and H/M ratio. These findings are concordant with previous studies [11, 22]. On the other hand, in our study, LV diastolic dysfunction was observed in 75% (9/12) of patients with decreased myocardial uptake and they showed significantly higher LAD, lower septal e′, and higher septal E/e′ ratio as compared with normal myocardial [^123^I]-MIBG uptake patients. The myocardial SUVmax was significantly lower in patients with LV diastolic dysfunction than in patients with normal cardiac function. Moreover, the myocardial SUVmax showed a significant positive correlation with septal e′, and a significant negative correlation with septal E/e′ ratio. These findings might indicate that LV diastolic dysfunction is inversely related to myocardial [^123^I]-MIBG uptake in pheochromocytoma patients. Galetta et al. [23] reviewed the cardiovascular complications in pheochromocytoma patients and speculated that the following mechanism of LV diastolic dysfunction; The direct myocardial toxicity caused by catecholamine exposure might lead to a form of myocarditis and produced the LV remodeling. The catecholamine-induced LV remodeling represented such as the collagen deposition with myocardial fibrosis, and the myocardial fibrosis adversely influenced diastolic stiffness of the myocardium and facilitated diastolic dysfunction. Recent studies demonstrated that impaired myocardial sympathetic innervation, as assessed by the ^11^C-hydroxyephedrine PET imaging, was highly associated with diastolic dysfunction and myocardial fibrosis among patients with heart failure with preserved ejection fraction (HFpEF) [24, 25]. HFpEF is a common form of heart failure and is functionally characterized by diastolic dysfunction accompanying myocardial fibrosis, and myocardial sympathetic denervation may reflect the pathophysiological process in HFpEF [26, 27]. Thus, we considered that the inverse relationship between the myocardial [^123^I]-MIBG uptake and LV diastolic dysfunction might be observed in pheochromocytoma patients. Although we found no significant difference in the H/M ratio between the groups with LV diastolic dysfunction and normal cardiac function, this might be explained by the fact that the H/M ratio was obtained on a planar image and might not provide sufficient anatomical information to enable an accurate assessment of myocardial [^123^I]-MIBG uptake.

Previous studies reported that in patients with neuroadrenergic tumors, myocardial [^131^I]-MIBG or [^123^I]-MIBG uptake was reduced and inversely correlated with circulating noradrenaline and adrenaline levels [11, 28]. In our analysis of 18 pheochromocytoma patients, neither significant difference in circulating noradrenaline and adrenaline levels between the decreased and normal myocardial uptake patient groups, and nor significant negative correlation between [^123^I]-MIBG myocardial uptake with circulating noradrenaline and adrenaline levels were observed, although plasma dopamine (the precursor of noradrenaline) levels showed a significant difference between these 2 groups and a significant negative correlation with the H/M ratio. This lack of significance might be due to the small study population and limited data of only pheochromocytoma patients. In fact, among 35 patients including 18 pheochromocytoma and 17 nonpheochromocytoma patients, myocardial visual score, myocardial SUVmax and H/M ratio were significantly lower in the pheochromocytoma patients than in the nonpheochromocytoma patients (supplemental Table 2) and each myocardial [^123^I]-MIBG uptake parameter showed significant negative correlation with plasma catecholamine levels except for plasma adrenaline levels (supplemental table 3). The lack of significant correlation between plasma noradrenaline or adrenaline levels and myocardial [^123^I]-MIBG uptake was also reported in patients with idiopathic dilated cardiomyopathy [29].

The denervated myocardium has not only been observed in patients with catecholamine-related myocarditis but also in patients with other cardiomyopathy including ischemic cardiomyopathy [30], idiopathic dilated cardiomyopathy [31] and hypertrophic cardiomyopathy [32]. As mentioned above, the relationship between the myocardial sympathetic denervation and diastolic dysfunction has been reported in patients with HFpEF as having cardiomyopathy including ischemic cardiomyopathy, idiopathic dilated cardiomyopathy and hypertrophic cardiomyopathy [24]. Thus, the presented inverse relationship between the myocardial [^123^I]-MIBG uptake and LV diastolic dysfunction might not be specific for the pheochromocytoma patients. Moreover, the gold standard imaging modality for diagnosis of LV diastolic dysfunction might be echocardiography, and the [^123^I]-MIBG scintigraphy might provide less additional information for diagnosis of LV diastolic dysfunction. However, to our knowledge, no studies have been examined the relationship between the myocardial sympathetic denervation assessed by the myocardial [^123^I]-MIBG SUVmax and LV diastolic dysfunction in pheochromocytoma patients. According to the presented results, the [^123^I]-MIBG scintigraphy using visual assessment and quantitative parameters, especially myocardial [^123^I]-MIBG SUVmax might have the potential to predict LV diastolic dysfunction in pheochromocytoma patients, and our results might suggest the complemental role of [^123^I]-MIBG scintigraphy for diagnosis of LV diastolic dysfunction in pheochromocytoma patients.

To the best of our knowledge, no previous studies have investigated the relationship between pheochromocytoma [^123^I]-MIBG uptake and cardiac function nor between pheochromocytoma [^123^I]-MIBG uptake and myocardial [^123^I]-MIBG uptake.

Our study did not observe a significant difference in the pheochromocytoma SUVmax between the normal cardiac function and LV diastolic dysfunction groups nor between the normal and decreased myocardial uptake groups. There were no significant correlations in echocardiographic parameters or myocardial SUVmax with the pheochromocytoma SUVmax. Moreover, we did not observe any significant correlations between pheochromocytoma SUVmax and catecholamine levels. These findings might indicate that neither secretion of catecholamine nor cardiac function is related to the degree of [^123^I]-MIBG uptake in pheochromocytoma.

In our study, the mean age was significantly higher in the LV diastolic dysfunction than in normal LV function groups. This result might suggest that cardiac sympathetic nerve function may be damaged more prolongedly in the older pheochromocytoma patients than in the younger pheochromocytoma patients by excess circulating norepinephrine. Age has been also reported to be associated with ventricular stiffness in HFpEF patients [33, 34].

This study had several limitations that warrant discussion. First, the study sample population was too small to reach a definitive conclusion. Therefore, there is a need for a prospective study with a much larger study population. Second, we examined the relationship only between pretreatment myocardial [^123^I]-MIBG uptake and cardiac function as assessed by echocardiography and did not evaluate the relationship between the two after treatment. Third, differences in the acquisition time after [^123^I]-MIBG injection can affect the visual or quantification analytical results. Myocardial [^123^I]-MIBG scintigraphic studies in heart failure are typically performed 10–30 min (early images) and 3–4 h (delayed images) after [^123^I]-MIBG is administered [18]. However, in the present study, we obtained the [^123^I]-MIBG cardiac planar and SPECT/CT images only 24 h after radiotracer injection because all patients underwent [^123^I]-MIBG scintigraphy for evaluation of adrenal tumors. Suga et al. [11] also analyzed the relationship between 24 h myocardial [^123^I]-MIBG uptake using H/M ratio and cardiac function in patients with neuroadrenergic tumors (pheochromocytomas and neuroblastomas). Fourth, we did not examine the usefulness of other semiquantitative [^123^I]-MIBG parameters including SUVmean for characterizing cardiac function. We did not use the SUVmean because, when the myocardium is poorly visualized, the generation of VOIs to calculate the SUVmean using threshold-based segmentation (e.g., 40% of SUVmax) can be unreliable and less reproducible. On the other hand, in spite of 24 h late [^123^I]-MIBG scintigraphy, intra- and inter-observer agreements were excellent or perfect for each scintigraphic parameter, suggesting high reproducibility.

In conclusion, the myocardial [^123^I]-MIBG SUVmax may be useful for characterizing cardiac function in patients with pheochromocytoma.

## Supplementary Information

Below is the link to the electronic supplementary material.

Supplementary file1 (DOCX 238 KB)
