# Supplementary material for: Application of 123I-MIBG myocardial maximum standardized uptake value to characterize cardiac function in patients with pheochromocytoma: comparison with echocardiography
Source: Jpn J Radiol. 2022 Nov 28;41(4):437–48. doi: 10.1007/s11604-022-01365-z (PMC10066163; doi:10.1007/s11604-022-01365-z)
Supplement: Supplementary file 1 — Supplementary file1 (DOCX 238 KB) [file 11604_2022_1365_MOESM1_ESM.docx]

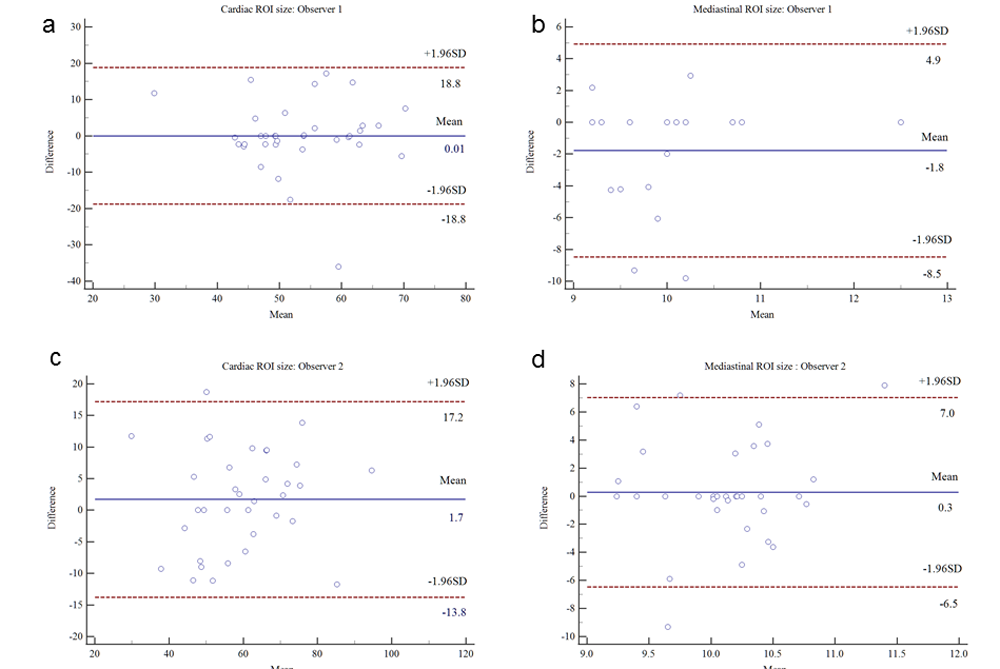


**Supplemental figure 1.** Intra-observer Bland-Altman plots of cardiac ROI size (a, c) and mediastinal ROI size (b, d) on planar [^123^I]-MIBG anterior images in 18 pheochromocytoma and 17 nonpheochromocytoma patients. Bland-Altman plots were used to analyze the agreement between the two evaluations per observer. The difference between two evaluations of one observer was plotted on the vertical axis and the mean of the two evaluations was plotted on the horizontal axis. The solid line represents the mean value for the data points and the dashed line represents the 1.96 × SD.


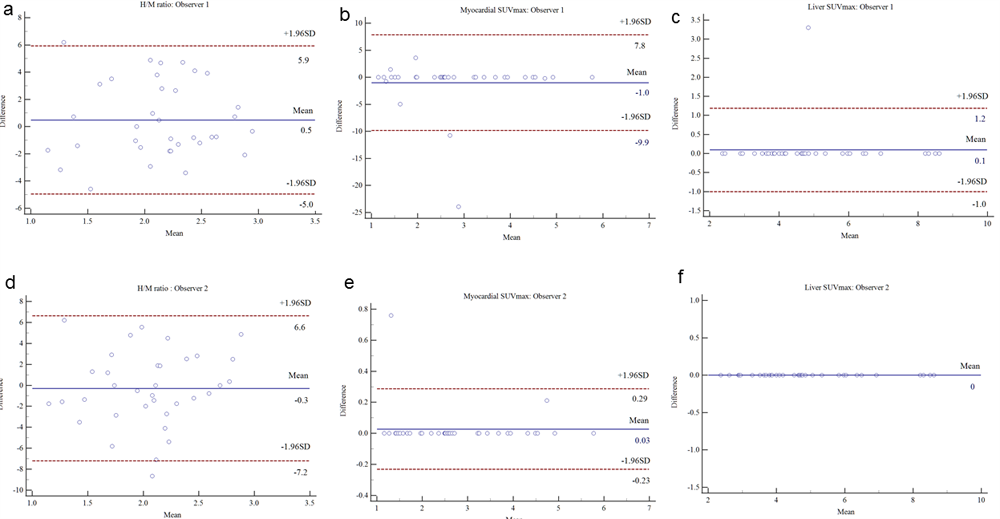


**Supplemental figure 2.** Intra-observer Bland-Altman plots of [^123^I]-MIBG H/M ratio (a, d), myocardial SUVmax (b, e), and liver SUVmax (c, f) in 18 pheochromocytoma and 17 nonpheochromocytoma patients. Bland-Altman plots were used to analyze the agreement between the two evaluations per observer. The difference between two evaluations of one observer was plotted on the vertical axis and the mean of the two evaluations was plotted on the horizontal axis. The solid line represents the mean value for the data points and the dashed line represents the 1.96 × SD.

| **Supplemental table 1.** The averaged cardiac and mediastinal ROI size for the first-time and second-time for observer 1 and observer 2 in 18 pheochromocytoma and 17 nonpheochromocytoma patients | | | | |
| --- | --- | --- | --- | --- |
|  | **Observer 1** | | **Observer 2** | |
|  | First time | Second time | First time | Second time |
| Cardiac ROI size (cm^2^) (mean, range) | 53.3 ± 9.1, 31.6–72.9 | 53.3 ± 9.1, 28.1–71.6 | 60.2 ± 14.0, 31.6–97.6 | 59.0 ± 13.2, 28.1–91.6 |
| Mediastinal ROI size (cm^2^) (mean, range) | 9.9 ± 0.7, 9.2–12.5 | 10.0 ± 0.6, 9.1–12.5 | 10.1 ± 0.5, 9.2–11.9 | 10.1 ± 0.5, 9.2–11.0 |

| **Supplemental table 2.** Comparison of [^123^I]-MIBG scintigraphic parameters between pheochromocytoma and nonpheochromocytoma patients | | | |
| --- | --- | --- | --- |
| Parameter | Pheochromocytoma Patients (n = 18) | Nonpheochromocytoma Patients (n = 17) | *p* value |
|  | Mean value ± SD (range) | Mean value ± SD (range) |  |
| Age | 58 ± 16 (28–76) | 66 ± 12 (40–83) | 0.15 |
| Catecholamine^*^ |  |  |  |
| Adrenaline (pg/mL) | 102.3 ± 90.5 (15.0–345.0) | 43.8 ± 51.8 (19.0–345.0) | 0.015 |
| Noradrenaline (pg/mL) | 1497.5 ± 1445.5 (233.2–5639.0) | 412.7 ± 211.1 (166.0–824.0) | <0.001 |
| Dopamine (pg/mL) | 91.7 ± 192.5 (6.0–701.3) | 13.8 ± 6.6 (5.0–26.0) | 0.035 |
| Liver SUVmax | 4.54 ± 1.8 (2.37–8.50) | 5.29 ± 1.6 (2.89–8.61) | 0.08 |
| Myocardial visual score | 1.3 ± 0.5 (1.0–2.0) | 2.0 ± 0.0 (2.0–2.0) | <0.001 |
| H/M ratio | 1.86 ± 0.45 (1.15–2.66) | 2.32 ± 0.32 (1.88–2.88) | 0.004 |
| Myocardial SUVmax | 2.35 ± 1.20 (1.16–4.75) | 3.28 ± 1.02 (2.27–5.77) | 0.009 |

| **Supplemental table 3.** Correlations of plasma catecholamine levels and [^123^I]-MIBG scintigraphic parameters in 35 pheochromocytoma and nonpheochromocytoma patients | | | | | | |
| --- | --- | --- | --- | --- | --- | --- |
| Parameter | Visual score | | Myocardial SUVmax | | H/M ratio | |
|  | Correlation | | Correlation | | Correlation | |
|  | ρ | *p* value | ρ | *p* value | ρ | *p* value |
| Catecholamine |  |  |  |  |  |  |
| Adrenaline | −0.22 | 0.22 | −0.06 | 0.72 | 0.04 | 0.83 |
| Noraderanaline | −0.45 | 0.007 | −0.44 | 0.009 | −0.50 | 0.003 |
| Dopamine | −0.53 | 0.001 | −0.44 | 0.001 | −0.49 | 0.003 |
| *SUVmax* maximum standardized uptake value, *H/M ratio*, heart-to-mediastinum ratio. | | | | | | |

| **Supplemental table 4.** Intra-observer agreement for [^123^I]-MIBG cardiac ROI size, mediastinal ROI size and [^123^I]-MIBG scintigraphic parameters with intraclass correlation coefficient and Bland-Altman analysis. | | | | |
| --- | --- | --- | --- | --- |
|  | **Observer 1** | | **Observer 2** | |
|  | Intraclass correlation coefficient^*^ | Bland-Altman analysis^†^ | Intraclass correlation coefficient^*^ | Bland-Altman analysis^†^ |
| Cardiac ROI size | 0.85 (0.68-0.91) | 0.01% (-18.8%, +18.8%) | 0.94 (0.88-0.97) | 1.7% (-13.8%, +17.2%) |
| Mediastinal ROI size | 0.86 (0.73-0.92) | -1.8% (-8.5%, +4.9%) | 0.86 (0.73-0.93) | 0.3% (-6.5%, +7.0%) |
| H/M ratio | 0.99 (0.99-0.99) | 0.5% (-5.0%, +5.9%) | 0.99 (0.97-0.99) | -0.3% (-7.2%, +6.6%) |
| Myocardial SUVmax | 0.99 (0.99-0.99) | -1.0% (-9.9%, +7.8%) | 1.00 (1.00-1.00) | 0.03% (-0.23%, 0.29%) |
| Liver SUVmax | 0.99 (0.99-1.00) | 0.1% (-1.0%, 1.2%) | 1.00 (1.00-1.00) | 0.0% (0.0%, 0.0%) |
| Pheochromocytoma SUVmax | 1.00 (1.00-1.00) | 0.0% (0.0%, 0.0%) | 1.00 (1.00-1.00) | 0.0% (0.0%, 0.0%) |
| ^*^ Numbers in parentheses are 95 % confidence intervals.  ^†^ The mean difference and in parentheses 95 % limits of agreement are shown. | | | | |

| **Supplemental table 5.** Inter-observer agreement between observers 1 and 2 for [^123^I]-MIBG cardiac ROI size, mediastinal ROI size and scintigraphic parameters with inter class correlation coefficient. | |
| --- | --- |
|  | Interclass correlation coefficient^*^ |
| Cardiac ROI size | 0.68 (0.36-0.84) |
| Mediastinal ROI size | 0.61 (0.23-0.80) |
| H/M ratio | 0.91 (0.84-0.96) |
| Myocardial SUVmax | 0.96 (0.92-0.98) |
| Liver SUVmax | 0.99 (0.98-0.99) |
| Pheochromocytoma SUVmax | 1.00 (1.00-1.00) |
| ^*^ Numbers in parentheses are 95 % confidence intervals. | |
